# Integrated Metabolomics and Network Pharmacology to Decipher the Latent Mechanisms of Protopanaxatriol against Acetic Acid-Induced Gastric Ulcer

**DOI:** 10.3390/ijms232012097

**Published:** 2022-10-11

**Authors:** Cuizhu Wang, Luying Tan, Juntong Liu, Dongxing Fu, Caixia Wang, Pingya Li, Zhuo Li, Jinping Liu

**Affiliations:** 1 School of Pharmaceutical Sciences, Jilin University, Changchun 130021, China; 2 Research Center of Natural Drug, Jilin University, Changchun 130021, China

**Keywords:** protopanaxatriol, gastric ulcer, metabolomics, network pharmacology

## Abstract

Gastric ulcer (GU) is a peptic disease with high morbidity and mortality rates affecting approximately 4% of the population throughout the world. Current therapies for GU are limited by the high relapse incidence and side effects. Therefore, novel effective antiulcer drugs are urgently needed. Ginsenosides have shown good anti-GU effects, and the major intestinal bacterial metabolite of ginsenosides, protopanaxatriol (PPT), is believed to be the active component. In this study, we evaluated the anti-GU effect of PPT in rats in an acetic acid-induced GU model. High (H-PPT) and medium (M-PPT) doses of PPT (20.0 and 10.0 mg/mg/day) significantly reduced the ulcer area and the ET-1, IL-6, EGF, SOD, MDA and TNF-α levels in serum were regulated by PPT in a dose-dependent manner. We also investigated the mechanisms of anti-GU activity of PPT based on metabolomics coupled with network pharmacology strategy. The result was that 16 biomarkers, 3 targets and 3 metabolomic pathways were identified as playing a vital role in the treatment of GU with PPT and were further validated by molecular docking. In this study, we have demonstrated that the integrated analysis of metabolomics and network pharmacology is an effective strategy for deciphering the complicated mechanisms of natural compounds.

## 1. Introduction

Gastric ulcer (GU) is a painful lesion of the gastric mucosa in the upper abdomen that can lead to more serious symptoms including internal hemorrhage, stenosis, perforation and pyloric obstruction. As a serious health problem affecting 4% of the population throughout the world [1], GU is a complex multicausal disease induced by multiple factors, including Helicobacter pylori infection, nonsteroidal anti-inflammatory drugs (NSAIDs), stress, alcohol, damaged barrier effect of the gastric mucosa, excess secretion of gastric acid, etc. [2,3]. The restoration of protective layers of the gastric mucosa and decreasing gastric acid secretion are important for minimizing the tissue damage in treating GU. However, the conventional medications in GU treatment, including proton pump inhibitors (PPIs), histamine H2 receptor blockers, Helicobacter pylori eradication therapy and antacids [4], may result in high relapse incidence and various side effects such as functional mucosal changes, intestinal microflora disorders, diarrhea and arrhythmia. Detecting efficient natural agents against GU may offer a chance to avoid the side effects of conventional therapies [5]. Ginsenosides are the principal active ingredients in Panax ginseng C. A. Meyer and are mainly responsible for its various pharmacological activities including protective effects against gastric ulcer [6,7,8]. Protopanaxatriol (PPT) is an aglycone of protopanaxatriol-type ginsenosides (Re, Rg1, Rg4, Rg6, Rh4, Rh1, Rg2, etc.), which were reported to possess wound-healing, anti-inflammation, immune-stimulatory and antioxidant activities in vivo in rats and mice [9,10,11]. Protopanaxatriol-type ginsenosides could be metabolized to PPT both in the stomach (acid hydrolysis) and in the gastrointestinal tract (bacterial hydrolysis) after oral administration [12,13,14]. In addition, PPT is not only a metabolite in body but also a natural compound that could be prepared by acid hydrolysis, alkaline hydrolysis or microbial transformation [15]. PPT has shown anti-oxidation and anti-inflammation activities [16,17]; however, there was no literature on the therapeutic effect of PPT on GU.

Metabolomics, defined as the comprehensive qualitative and quantitative analysis of all small molecules (metabolites) present in a biosystem [18], is a branch of “omics” that involves the identification and quantification of metabolites and chemical footprints of cellular regulatory processes in different biological species [19] to monitor the dynamic changes in endogenous metabolites and systematically link metabolic pathways and disease processes as well as elucidate the mechanisms of action (MOA) of drugs [20]. Network pharmacology, first proposed by Hopkins in 2008, emphasizes ‘multi-compounds, multi-target, and multi-disease’ rather than ‘a-drug, a-gene, a-disease’ drug action patterns and appropriately adheres to the holistic approach of herbal medicines [21,22], which is used to explore the complex relationships among proteins, diseases and drugs [23]. A strategy of integrating these two approaches offers the opportunity to elucidate the molecular mechanism by connecting active ingredients, targets, endogenous metabolites and pathways, which may compensate each other for upstream molecular mechanisms and pathophysiological data.

In the current study, an acetic acid-induced gastric ulcer model, one of the representative experimental GU models [24,25], was used to investigate the therapeutic effect of PPT for treating GU. We also developed an integrated strategy combining metabolomics and network pharmacology to elucidate the molecular mechanism of PPT’s anti-GU effects; then, we used molecular docking to show the high affinities between key targets and PPT. The results shed light on the complicated pathogenesis of GU and suggested a potential natural anti-GU agent and strategies.

## 2. Results

### 2.1. Anti-GU Effect

#### 2.1.1. Body Weights of the Rats

**Figure 1:**
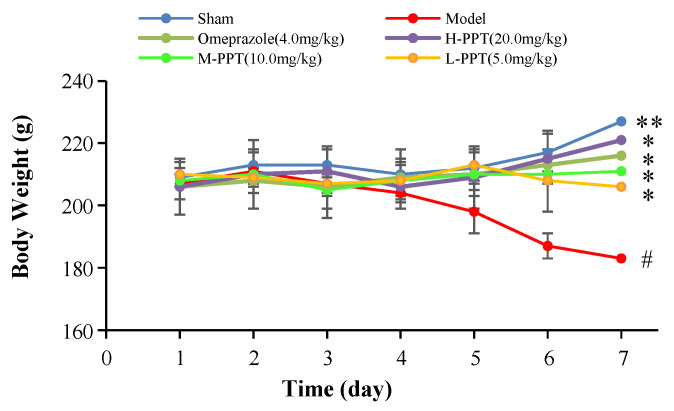
Body weights of animals (compared with sham group, # *p* < 0.05; compared with model group, * *p* < 0.05, ** *p* < 0.01).

The body weights of the rats in all groups, i.e., high-dose PPT (H-PPT, 20.0 mg/kg), moderate-dose PPT (M-PPT, 10.0 mg/kg), low-dose PPT (L-PPT, 5.0 mg/kg), sham, model and positive (omeprazole, 4.0 mg/kg), were measured every day, and the results are shown in Figure 1. Over seven days, the rats in the model group had a significant weight loss, while the body weights of rats in omeprazole, M-PPT and H-PPT increased gradually. In addition, although the body weights of rats in L-PPT group increased on the 4th and 5th days, the body weights began to decrease from the 6th day. On the 7th day, compared with the sham group, the body weights in the model group significantly decreased (*p* < 0.01). However, compared with the model group, the intervention of all doses of PPT or omeprazole could significantly alleviate the weight loss caused by gastric ulcer (*p* < 0.05).

#### 2.1.2. Macroscopic Results

**Figure 2:**
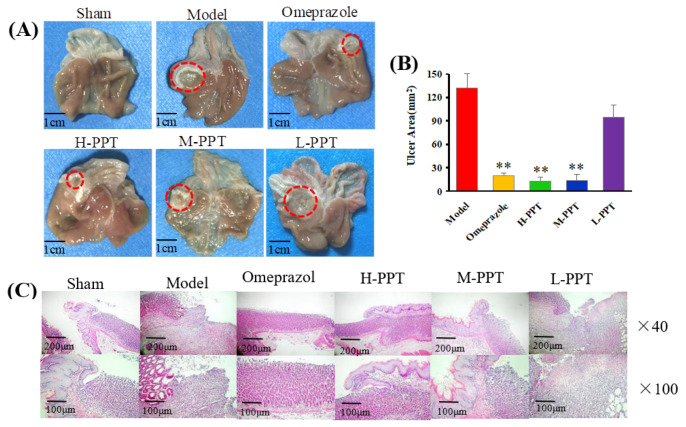
Macroscopic and microscopic pictures of the stomachs of all groups demonstrated the validity of the model employed. (**A**) The macroscopic images of stomachs from all groups. The red circle indicates ulcer location. Scale bar = 1 cm. (**B**) The ulcer areas of different groups (compared with the model group, ** *p* < 0.01). (**C**) Hematoxylin and eosin (H&E) staining of the gastric mucosa.

In order to evaluate the ulcer formations, the macroscopic appearances of stomachs were captured, and the ulcer area was calculated. The macroscopic appearances of stomachs in different groups were captured: Macroscopic images of stomachs are shown in Figure 2A, and the ulcer area measurements are presented in Figure 2B. The rats in the sham group received 0.9% saline solution and displayed no gastric lesions. Compared with the model group (133.48 ± 8.42 mm^2^), the omeprazole group (19.63 ± 2.42 mm^2^), H-PPT group (18.09 ± 3.54 mm^2^) and M-PPT group (21.23 ± 5.61 mm^2^) had significantly reduced ulcer areas. However, L-PPT (94.99 ± 10.27 mm^2^) showed no significant difference compared with the model group.

#### 2.1.3. Histological Evaluation of Gastric Ulcers

The general microstructures of the gastric mucosa were observed using H&E staining. As shown in Figure 2C, the normal stomach tissue (serosa, muscularis, mucosa and submucosa) was healthy without symptoms such as hemorrhage necrosis, glandular area hyperemia or mucosal edema. In the model group, the epitheliums lost their integrity, and several symptoms such as mucosa edema, mucosa lesions, large ulcers, gland hyperemia and infiltrated inflammatory cells were observed. The H-PPT, M-PPT and omeprazole alleviated the gastric mucosal injuries, while the L-PPT group showed little improvement of the GU-related histopathologies. Thus, the preventive effects of PPT on gastric mucosa were enhanced with increased concentration.

#### 2.1.4. ET-1, IL-6, EGF, SOD, MDA and TNF-α Levels in Serum

**Figure 3:**
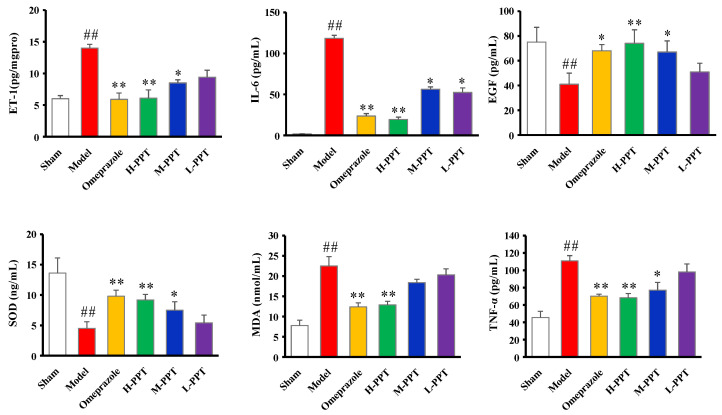
Biochemical estimations in serum evaluated the effects of different groups against GU. The ET-1, IL-6, EGF, SOD, MDA and TNF-α levels in serum. (Compared with the sham group, ## *p* < 0.01; compared with the model group, * *p* < 0.05, ** *p* < 0.01).

The biochemical estimations in serum including ET-1, IL-6, EGF, SOD, MDA and TNF-α levels were carried out according to the instructions provided by the manufacturers. As shown in Figure 3, compared with the sham group, serum ET-1, IL-6, MDA and TNF-α levels significantly increased in acetic acid-induced GU rats, while EGF and SOD levels decreased. PPT re-regulated the ET-1, IL-6, EGF, SOD, MDA and TNF-α levels in a dose-dependent manner, and H-PPT showed a similar effect to the positive omeprazole.

#### 2.1.5. Immunohistochemical Analysis

**Figure 4:**
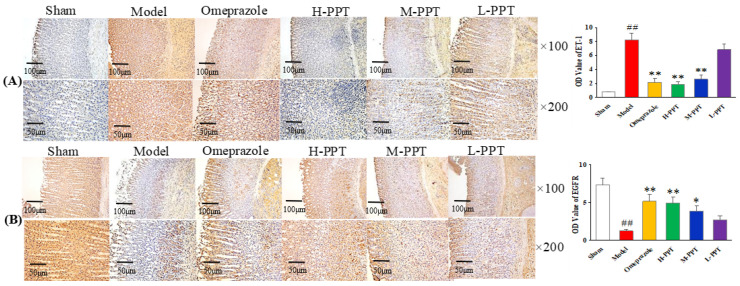
Photomicrography of histological sections and the corresponding OD values to determine the protein expressions in gastric mucosa. (**A**) ET-1 staining cells and (**B**) EGFR staining cells in different groups (×100 and ×200). (Compared with sham group, ## *p* < 0.01; compared with model group, * *p* < 0.05, ** *p* < 0.01). (OD value: optical density value).

The expressions of ET-1 and EGFR in gastric mucosa were analyzed, by calculating the OD value. As shown in Figure 4A, in the immunohistochemical analysis, the ET-1 levels in the model group increased markedly compared with the sham group (*p* < 0.01), while the expressions of ET-1 in H-PPT and M-PPT rats decreased to a similar extent as in the omeprazole group, suggesting that PPT treatment reduced the expression of pro-inflammatory cytokine. The level of EGFR significantly decreased in the model group (*p* < 0.01) (Figure 4B). PPT could increase the levels of EGFR in a dose-dependent manner: specifically, a higher dose corresponded to a better effect. H-PPT showed a similar effect to omeprazole in the improvement of gastric mucosa integrity and restrictions of systemic inflammation.

### 2.2. Metabolomics Study

#### 2.2.1. Method Validation

Sixteen ions were randomly selected from QC sample for the method validation throughout the whole study. The ions with the retention times and mass-to-charge ratios (*m*/*z*) from different spectral regions were as follows: 0.78–276.1187, 1.51–188.0742, 5.04–582.2692, 10.35–874.4197, 14.31–415.2157, 16.58–520.3442, 17.95–496.3475, 23.12–413.2453 in ESI+ mode, 0.52–197.8073, 3.57–241.1203, 8.77–225.1245, 12.44–453.2865, 16.96–564.3336, 20.55–568.3600, 22.79–303.2343, 24.95–281.2469 in ESI- mode. The RSDs of the peak intensity and RT in system stability, precision, reproducibility and sample stability tests are shown in Table 1.

#### 2.2.2. Identification of the Differential Metabolites and Pathway Analysis

In order to identify the differential metabolites and related metabolic pathways, multivariate data analysis including PCA and OPLS-DA was performed, and ROC curves were generated. The metabolites with *p* value below 0.05, VIP above 1.0 and AUC above 0.8 were considered potential biomarkers and further identified by standards or MS/MS fragmentation. Potential metabolites and metabolic pathways were confirmed by MetaboAnalyst 5.0.

**Figure 5:**
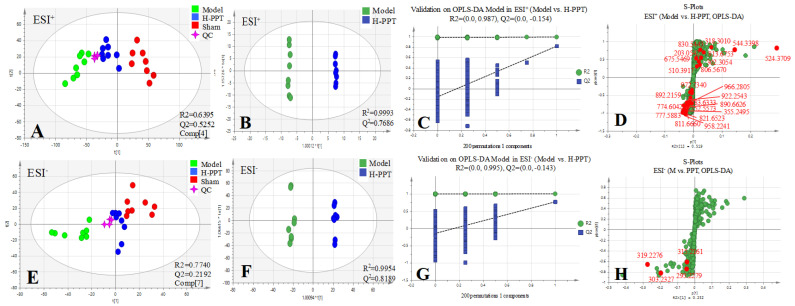
Multivariate statistical analysis resulting from rat serum. (**A**,**E**) PCA score plots of serum metabolic profiling of sham, model and H-PPT groups in positive and negative modes; (**B**,**F**) OPLS-DA score plots of model and H-PPT groups in positive and negative modes; (**C**,**G**) the permutations plots of the OPLS-DA models in positive and negative modes; (**D**,**H**) S-plots of serum metabolic profiling in positive and negative modes.

**Figure 6:**
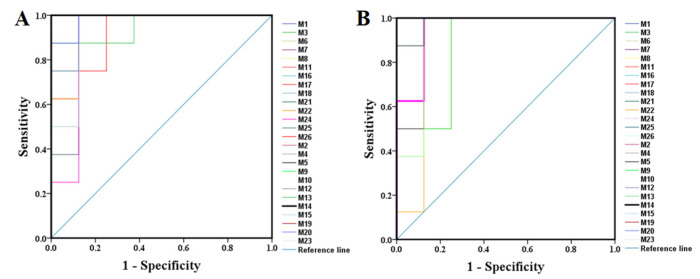
The predictive ROC curves generated using 26 biomarkers contributing to GU progress (**A**) and PPT treatment (**B**) (the numbers are consistent with No. in Table 1).

**Figure 7:**
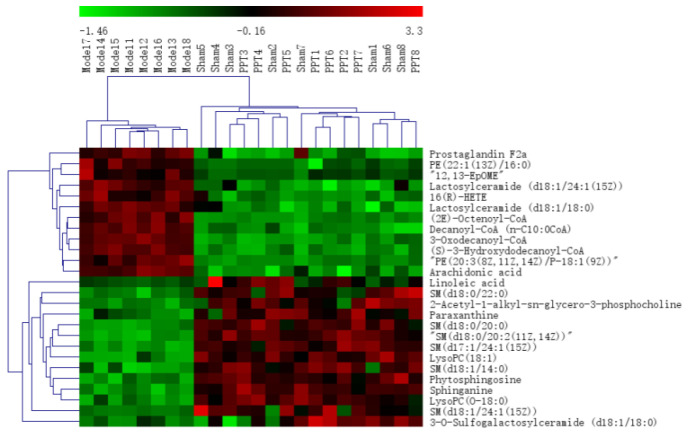
The heatmap of the potential metabolites.

All the selected samples were analyzed by the validated UPLC-QTOF-MS method. The superimposed view of the representative base peak intensity (BPI) chromatograms in positive and negative modes of the three groups of serum samples is shown in Figure S1 in the Supplementary Materials. Pareto scaling was performed to generate the PCA, OPLS-DA and S plots. As shown in Figure 5A,E, PCA showed a clear separation of three groups where the samples from the same group were generally clustered together. In addition, the QC samples were clustered together in the middle, showing good stability and reproducibility. To maximize the separation among the different groups and identify potentially differentially regulated metabolites, OPLS-DA was used to compare the metabolomics data between the H-PPT treatment group and the model group in both ESI+ and ESI- modes (Figure 5B,F). A clear separation between groups was also observed with satisfactory parameters (R2 and Q2), suggesting good reliability and prediction ability. Then the permutation test with 200 iterations was used to access the quality of the OPLS-DA model (Figure 5C,G). The results indicated that the models were not overfitted because the original points to the right were higher than the Q2-values to the left. The S-plots based on the OPLS-DA model were generated to identify differentially abundant metabolites contributing to the separation. In S-plots, a longer distance between the origin and the spot represented more contribution to the discrimination between H-PPT group and the model group. A total of 27 metabolites with VIP > 1 and *p* < 0.05 were considered as preliminary potential biomarkers. The predictive ROC curves were constructed using the 27 potential metabolites. The ROC curve between the sham group and the GU group indicated that all 27 metabolites were potential diagnostic markers for GU. The ROC curve between the GU group and the H-PPT group indicated that the potential biomarkers contributed to the effect of PPT on GU treatment. Following up, 26 metabolites with the area under curve (AUC) value > 0.8 were identified as the biomarkers and marked in red in S-plots (Figure 5D,H). The AUC values and *p* values of the biomarkers in ROC curves are listed in Figure 6 and Table 2 and detailed information of the biomarkers were listed in Table 3. The changes of each biomarker in different groups were shown in Figure S2 in Supplementary Materials, and the heatmap was used to characterize the relative abundance of the identified metabolites in three groups (Figure 7), with the color from green to red, the relative abundance of the biomarkers goes higher.

By MetaboAnalyst analysis, the identified biomarkers were mainly involved in 7 potential metabolisms with impact values above 0.10 (Table 4). Alterations of metabolites of the H-PPT group was mapped to caffeine metabolism (CM), glycerophospholipid metabolism (GM), sphingolipid metabolism (SM), fatty acid elongation (FAE), arachidonic acid metabolism (AM), linoleic acid metabolism (LM) and ether lipid metabolism (EM).

### 2.3. Network Pharmacology

To elucidate the interactions between PPT and anti-GU activity, and predict the related potential targets and pathways from a comprehensive perspective, network pharmacology was performed. The disease targets of GU and molecular targets of PPT were searched and filtered using various databases, and their intersection was considered the predicted targets of PPT against GU. Then, GO and KEGG analysis were performed, and the PPI network and component-target-pathway networks were established.

A total of 119 targets of PPT and 7170 targets related to GU were retrieved from various databases. The PPT-related targets were matched with the candidate targets of GU; specifically, 89 common targets were identified (Table S1 in the Supplementary Materials), which were considered the core targets of the following research. As shown in Figure S3, the PPT-target-disease network was constructed with 91 nodes and 358 edges using Cytoscape 3.8.2 software.

**Figure 8:**
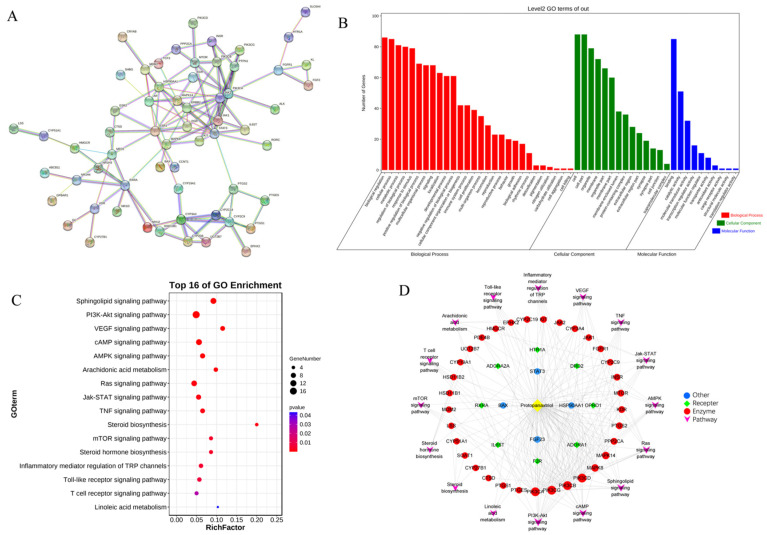
The network pharmacological analysis. (**A**) The PPI network. (**B**) GO enrichment histogram. (**C**) Bubble chart of KEGG enrichment. (**D**) The ‘component-target-pathways’ network.

To identify the hub genes of PPT against GU, a protein–protein interaction (PPI) network was constructed (Figure 8A) to show an overview of the relationship within 73 targets (the other 16 genes were disconnected). Based on PPI topology analysis, hub genes were filtered by mean degree (degree ≥ 14), and then the hub gene network (Figure S4) was constructed using CytoHubba. The color depth of the network nodes was positively correlated with the degree. The darker colors of CYP2C9, STAT3, JAK1, MAPK8, RXRA, IL6ST, ERBB2, CYP3A4, PIK3CA and JAK2 indicated that they were more important in the network. GO enrichment analysis yielded 52 GO entries including 28 biological process (BP), 14 cellular component (CC) and 10 molecular function (MF) (Figure 8B). The bubble chart of KEGG enrichment is shown in Figure 8C. The size of the bubbles in the figure was proportional to the number of genes, and the color of the bubbles was affected by the *p* value. The abscissa represented the enrichment score, while the ordinate represented signal pathways. The signal pathways such as the sphingolipid signaling pathway and the PI3K-Akt signaling pathway corresponded to more target genes, suggesting that these pathways may be important pathways for PPT to exert anti-GU effects. Interestingly, sphingolipid metabolism, arachidonic acid metabolism and linoieic acid metabolism were significantly enriched, which was partly consistent with the results of serum metabolomics analysis. As shown in Figure 8D, the ‘component-target-pathways’ network was established based on the KEGG bubble chart. The network contains 61 nodes and 238 edges. PPT was connected to multiple targets, indicating that PPT can activate multiple targets. In addition, multiple pathways in the network were connected by some common targets, rather than separated independently, indicating that the involved pathways were interconnected and may act synergically.

### 2.4. Integrated Analysis of Metabolomics and Network Pharmacology

**Figure 9:**
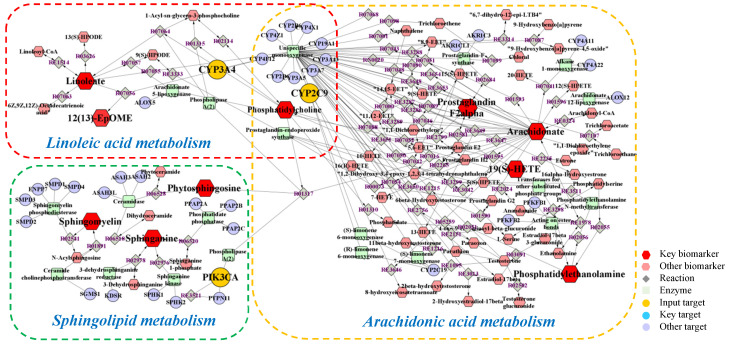
The compound-reaction-enzyme-gene networks of the key metabolites and targets. The red hexagons, grey diamonds, green round rectangle, purple circles and orange circles represent the active compounds, reactions, proteins, genes and hub genes, respectively. The key metabolites, proteins and genes were magnified.

Metabolomics and network pharmacology were merged to construct an integrative network, obtaining a comprehensive view of the mechanism of PPT against GU. The potential biomarkers and metabolic pathways identified in metabolomic study and the potential hub targets and pathways identified in network pharmacology were imported into the MetScape plugin in Cytoscape 3.8.2 software to generate the ‘compound-reaction-enzyme-gene’ networks (Figure 9).

To sum up, a total of 16 biomarkers (19(S)-HETE, arachidonic acid, prostaglandin F2a, linoleic acid, 12,13-EpOME, phytosphingosine, sphinganine, PE(22:1(13Z)/16:0), PE(20:3 (8Z,11Z,14Z)/P-18:1(9Z)), PC(O-16:0/2:0), SM(d18:0/22:0), SM(d18:0/20:0), SM (d18:0/20:2 (11Z,14Z)), SM(d17:1/24:1(15Z)), SM(d18:1/14:0), SM(d18:1/24:1(15Z))), 3 key targets (CYP2C9, CYP3A4, PIK3CA), and 3 metabolic pathways (arachidonic acid metabolism, linoleic acid metabolism and sphingolipid metabolism) were confirmed, which were considered to play vital roles in the therapeutic effect of PPT on GU.

### 2.5. Molecular Docking

In order to predict the binding modes of key targets with PPT and investigate the localized binding sites and relative binding energies in the active pocket of key genes, molecular docking was carried out by retrieving the crystal structures of key targets, preparing the ligand and performing the GLIDE docking.

**Figure 10:**
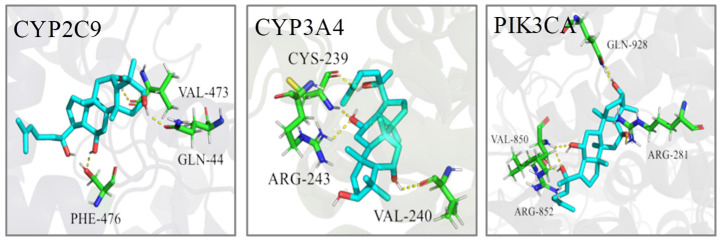
The 3D interaction diagrams of PPT and the key targets.

The PDB IDs of the protein structures we employed are displayed in Table 4. The molecular docking calculation predicted the most probable structural configurations and binding sites (Figure 10). PPT formed three hydrogen bonds with VAL-473, GLN-44 and PHE-476 residues in CYP2C9 while PPT interacted with CYP3A4 through hydrogen-bonding with CYS-239, ARG-243 and VAL-240. There were six hydrogen bonds formation between PPT and PIK3CA involving four residues including GLN-928, VAL-850, ARG-281 and ARG-852. The binding energies of PPT towards the three targets were −6.78, −9.40 and −9.27 Kcal/mol, respectively (Table 5), suggesting the high affinities of PPT with the key targets.

## 3. Discussion

Gastric ulcer (GU) is an ulcerative lesion in the surface of stomach mucosa, which is caused by various invasive factors especially the gastric acid secretion. Conventional medications to treat GU have shown various side effects and high relapse incidence. Thus, an effective antiulcer drug with few side effects is needed. PPT as an important human microbial metabolite of ginsenosides has shown good anti-oxidation and anti-inflammation activities. In this study, we have evaluated the therapeutic effect of PPT on GU.

GU is one of the major gastrointestinal disorders, which often occur due to an imbalance between offensive factors and defensive factors [26]. The offensive factors mainly include gastric acid secretion, pepsin, proinflammatory cytokines (such as IL-6, TNF-α, ET-1, IL-2, IL-1β), oxidative stress (such as MDA, SOD, NOS2, GSH-Px) and platelet activating factor (PAF), and the defensive factors mainly include gastric mucus, gastric mucosa, EGF and EGFR, NO, NOS and prostaglandins (PGs). In addition, various evidence has indicated that both inflammation and oxidative stress played an important role in GU. ET-1, IL-6 and TNF-α were vital pro-inflammatory cytokines in GU formation. ET-1 could contract the vessels, reduce the blood supply of gastric tissue and cause local hypoxia and acidosis [27,28]. IL-6 could induce tissue damage by initiating neutrophil accumulation, inducing a transcriptional inflammatory response and producing noxious products, reactive oxygen radicals and the lysosomal enzymes responsible for tissue damage in GU [29,30]. TNF-α could elicit an acute inflammatory reaction accompanied by neutrophil infiltration in gastric mucosa and also regulate apoptotic cell death in the gastric mucosa [31]. SOD could convert superoxide to hydrogen peroxide, and MDA is the final product of lipid peroxidation that causes loss of membrane integrity. Both SOD and MDA were reported to play important roles in the formation and development of the ulcer [32]. EGF is a ligand of EGFR that reduces gastric acid secretion and promotes the epithelial cell repair. EGF can aid the healing of ulcers when secreted in the gastrointestinal tract, increase the blood flow of the basic gastrointestinal mucosa and regulate the immune system of the digestive tract [33]. EGFR is important for the proliferation and differentiation of gastric mucosal cells, and its expression increases during early stages of gastric ulcer healing [34]. Thus, the proinflammatory cytokines including IL-6 and TNF-α and reactive oxygen species including MDA and SOD, together with ET-1, EGF and EGFR, were used in this study to evaluate the anti-GU effect of PPT. In the acetic acid-induced GU model, SOD, EGF and EGFR were down-regulated, while ET-1, IL-6, MDA and TNF-α were up-regulated. The intervention of PPT treatment re-regulated these factors to normal levels and significantly reduced the ulcer areas in a dose-dependent manner, suggesting a remarkable gastroprotective effect of PPT.

Metabolomics and network pharmacology analysis were carried out to identify the pharmacological effect from the aspects of potential biomarkers, metabolic pathways, targets, respectively. Firstly, metabolomics was used to decipher the dynamic changes of endogenous metabolites for assessing drug effects systematically. In our study, pattern recognition with multivariate statistical analysis suggested that the metabolic profiles of the acetic acid-induced GU rats were markedly different from that of the normal control rats, while the PPT-treated GU treatment groups had similar metabolomic profiles as the normal control group. We identified 26 potential metabolites involving 7 metabolic pathways. Secondly, network pharmacology at the molecular level was carried out to explain the relationships between the latent metabolites and pathways. The PPI network was constructed, the bub genes were screened out, and the component-target-pathway network indicated that PPT was closely related with 45 target genes and 16 pathways. Finally, an integrated analysis of metabolomics and network pharmacology was performed, and the ‘compound-reaction-enzyme-gene’ network was established: 16 biomarkers, 3 targets and 3 metabolic pathways were screened out that were considered to be closely related to the therapeutic effect of PPT on GU.

Two of these three pathways were lipid metabolism, including linoleic acid metabolism and sphingolipid metabolism, and one was arachidonic acid metabolism. Alteration in the metabolism of lipid was reported to be disturbed in GU [35]. Phosphatidylcholines (PCs) as the most abundant phospholipid in the cell and are directly related to cell proliferation, also play a vital role in lipid metabolism. Elevated level of 12,13-EpOME and decreased level of linoleic acid and PC (O-16:0/2:0) were observed in GU rats and restored by PPT as compared with the controls, suggesting PPT might relieve GU by stimulating linoleic acid metabolism, in accordance with previous studies [36]. Sphingolipid metabolism was also reported to participate in the occurrence and development of ulcer [37]. Sphingomyelins (SMs) are a group of common mammalian cell membrane sphingolipids that maintain cellular stability and regulate signal transduction. SMs including SM (d18:0/22:0), SM (d18:0/20:0), SM (d18:0/20:2 (11Z,14Z)), SM(d17:1/24:1(15Z)), SM(d18:1/14:0), SM (d18:1/24:1(15Z)) were down-regulated in the GU group. Phytosphingosine can prevent the acute ulcer formation while sphinganine participates in regulating the cell growth, adhesion, migration, death and inflammation process [38,39]. The abovementioned metabolites were re-regulated to normal levels after PPT treatment in GU rats, which highlights the involvement of sphingolipid metabolism in GU. In the arachidonic acid metabolism, arachidonic acid is one constituent of the cell membrane, which can be hydrolyzed, released or metabolized to various bioactive substances such as prostaglandins or 19(S)-HETE [40]. The levels of 19(S)-HETE, arachidonic acid and prostaglandin F2a elevated in the GU model group. Phosphatidylethanolamines (PEs) are a kind of gastricsurface active phospholipids which are synthesized and secreted in the gastric surface cells [41]. In the model group, increased levels of PE(22:1(13Z)/16:0), PE(20:3(8Z,11Z,14Z)/P-18:1(9Z)) and LysoPC (18:1(9Z)) were observed. They were re-regulated after PPT treatment indicating that the imbalance of arachidonic acid metabolism in GU can be reversed by PPT.

Three targets including CYP2C9, CYP3A4 and PIK3CA were suggested as the potential therapeutic targets. CYPs are members of the cytochrome P450 families, which are mainly expressed in the gastrointestinal tract and human liver. CYPs can convert numerous exogenous compounds, such as most therapeutic agents, to less toxic components [42,43]. CYP2C9 and CYP3A4 are two major CYP enzymes which are vital determinants for the oral bioavailability of drugs. CYP2C9 plays a key role in the oxidative metabolism of up to 15% drugs that undergo phase I metabolism while this enzyme was also closely related to non-aspirin NSAID-related gastrointestinal bleeding [44,45,46]. CYP3A4 is a dominant CYP enzyme highly expressed in both liver and extra-hepatic tissues. It metabolizes around 60% of currently used drugs [47,48]. Omeprazole and glycyrrhizin are the drugs that widely used to treat peptic ulcer. Co-administration of omeprazole and glycyrrhizin has shown better therapeutic effect which was believed to through inducing the activity of CYP3A4 [49]. Since the significant role of CYP3A4-mediated metabolism in PPIs clearance in humans, it is important to gain a better insights of the drug–drug interaction potential of PPIs with CYP3A4 inhibitors or inductors [50]. Patients with GU have high risk of developing gastric cancer [51]. Phosphatidylinostitol 3-kinase catalytic subunit (PIK3CA) can decrease the invasive capacity of gastric cancer cells in vitro [52]. PIK3CA amplification could activate the PI3K/Akt pathway in gastric cancer resulting in the poor survival rates of gastric cancer patients [53]. Microsomal cytochrome P450 also played an important role in the metabolism of PPT. It was reported that the oxidized metabolites of PPT were formed via combinatorial metabolism including both colonic microflora and the cytochrome P450 enzymes [54]. Both chemical inhibition and human recombinant P450 isoform assays indicated that CYP3A4 was the predominant isozyme responsible for the oxygenation metabolism of PPT ginsenosides [55]. PPT was metabolized to a pair of epimers (namely, M1-1 and M1-2), and CYP3A4 was the predominant isoform involved in the oxidative metabolism of M1-1 and M1-2 [56].

Cytochrom enzymes including CYP2CA and CYP3A4 are very important superfamily of biotransformation enzymes that are involved in oxidative metabolism of a wide variety of endogenous and exogenous compounds such as drugs. Numerous people in many countries have taken ginseng or its derived products, however, little is known about the interactions between ginseng and prescription drugs. Therefore, it is necessary to evaluate whether ginseng and its active components exhibit the potential to exert influence on metabolic enzymes. Thus, it is meaningful to explore the relationship between PPT and P450 enzymes. Actually, the complex relationship among PPT, biomarkers, targets and metabolic pathways were found based on metabolomics study combined with network pharmacology study. That is, some metabolites had been re-regulated after PPT binding to CYP3A4 and CYP2CA. Just as shown in Figure 10, PPT could indirectly regulate the metabolites such as linoleic acid and 12,13-EpOME by acting on the CYP2CA and CYP3A4. Although we did not perform the CYP induction assays, but previous studies showed that PPT exhibited moderate inhibition against CYP2C9 with an IC50 of 33.7 ± 2.7 μM, and inhibitory effect against CYP3A4 with an IC50 of 7.1 ± 0.9 μM [57]. The activity of cytochrome P450 enzymes generated reactive metabolites and free radicals which in turn bind to macromolecules, caused membrane lipid peroxidation, and increased cellular toxicity. PPT might also inhibit the activities of cytochrome P450 enzymes in order to attain dynamic homeostasis and reduce cellular toxicity [58].

Taken together, the three key target genes play important roles in the occurrence and development of gastric ulcer. Molecular docking also verified the high affinities of PPT with these key targets, while CYP3A4/PPT has the lowest binding energy.

Our findings showed that PPT could exert anti-GU effect similar to that of omeprazol. Though they showed similar effect, the mechanism of them were not the same. Omeprazol is belonging to proton pump inhibitors (PPIs), which could inhibit H+, K+-ATPase (proton pump), reduce the H+ secretion by parietal cells, effectively decrease gastric acid secretion and exert anti-GU effect. As for PPT, our study based on integrated metabolomics and network pharmacology showed that PPT could play a therapeutic effect on GU by regulating three key targets, 16 biomarkers and 3 metabolomic pathways. Both PPT and omeprazol showed good anti-GU effect, so they may be used together in patients. Though the mechanism of PPT and omeprazol were different, it is not sure whether there would be some potentially drug interactions after combination of them. Ginsenosides always showed synergistic effect by increasing efficacy and decreasing toxicity when combined with other drugs. For example, ginsenoside Rg3 can enhance drug efficacy and reduce drug-induced toxicity when used together with first-line chemotherapy drugs in clinical [59], ginsenoside compound K could enhance the efficacy of cisplatin in lung cancer cells with low toxicity and minimal side effects [60]. So we speculated that PPT may increase efficacy and decrease toxicity when combined with omeprazol against GU, which would be studied further in the future.

However, we are aware that our research may have some limitations. For instance, it would have been better if the experiments had been set up as days 1, 3, 5 and 7 and the data had been collected on each of these days. Future studies could improve the experimental design and collect time-course data.

## 4. Materials and Methods

### 4.1. Materials

Protopanaxatriol (CAS Number: 1453-93-6, purity > 98.0%) was provided by the Research Center of Natural Medicine of Jilin University (Jilin, China). Omeprazole was chosen as the positive drug and purchased from Sigma (St. Louis, MO, USA). Enzyme-linked immunosorbent assay (ELISA) kits of ET-1 and IL-6 were obtained from ImmunoWay Biotechnology Company (Plano, TX, USA). ELISA kits of Epidermal growth factor (EGF) was obtained from JiuBang Biotechnology Company (Quanzhou, China). ELISA kits of TNF-α were purchased from MultiSciences (Lianke) Biotech Co., Ltd., Hangzhou, China, and ELISA kits of SOD and MDA were bought from ZCIBIO Technology Co., Ltd., Shanghai, China. The antibodies of ET-1 and Epidermal growth factor receptor (EGFR) were purchased by Abcam (Cambridge, MA, USA). UPLC grade acetonitrile and methanol were bought from Fisher Chemical Company (Geel, Belgium). UPLC grade formic acid was purchased from Sigma-Aldrich (St. Louis, MO, USA). All other chemicals were used at the analytical grade. Deionized water was produced by a water purification system (Millipore, Billerica, MA, USA). Acetic acid was 36~38% and of analytical grade from J&K Technology Co., LTD. Chloral hydrate was provided by Biosharp Co., Ltd. (Shenyang, China). Paraxanthine was provided by Beijing Laiyao Biological Technology Co., Ltd. (Beijing, China). Linoleic acid and arachidonic acid were obtained from Sigma-Aldrich Co., Ltd., (St. Louis, MO, USA). Phytosphingosine and sphinganine were from Beijing Century Aoke Biological Technology Co., Ltd., Beijing, China. 12,13-EpOME was purchased from Shanghai Zhen Zhun Biological Technology Co., Ltd., Shanghai, China. 19(S)-HETE was manufactured from Xi’an Ruixi Biological Technology Co., Ltd., Xi’an, China.

### 4.2. Methods

**Figure 11:**
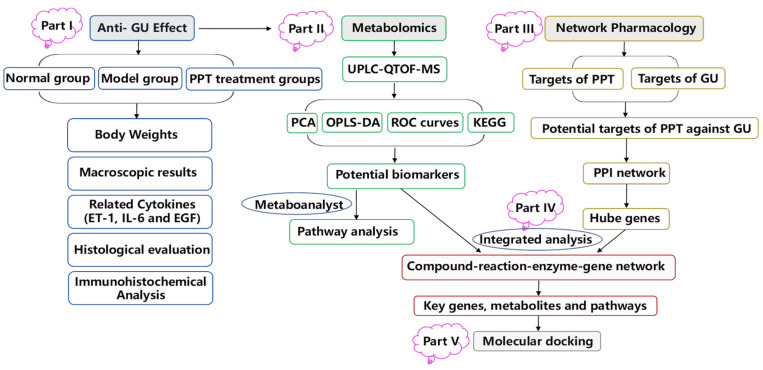
The schematic flowchart of the study.

In the study, an acetic acid-induced gastric ulcer model was used to investigate the therapeutic effect of PPT for treating GU, and an integrated strategy combining metabolomics and network pharmacology was used to elucidate the molecular mechanism of PPT’s anti-GU effects; then, molecular docking was used to show the high affinities between key targets and PPT. The research flowchart of the study is shown in Figure 11.

#### 4.2.1. UPLC-QTOF-MS Conditions

A Waters Xevo G2-XS QTOF mass spectrometer (Waters Co., Milford, MA, USA) equipped with a UPLC system by an electrospray ionization (ESI) interface was applied for analysis. Data recording was carried out on a MassLynx V4.1 workstation (Waters Co., Milford, MA, USA).

The optimized parameters of Q-TOF/MS were set as follows: drying gas, N2; desolvation temperature, 400 °C; capillary voltage, 2.6 kV (ESI+) and 2.2 kV (ESI−); desolvation gas flow, 800 L/h; mass, 50–1200 Da; cone voltage, 40 V; collision energy of high energy function, 20–40 V; collision energy of low energy function, 6 V. Leucine enkephalin (*m*/*z* 556.2771 in the positive mode and 554.2615 in the negative mode) was injected at a rate of 10 μL/min as external reference of Lock Spray™. Centroid mode was used to record the MassLynx data.

The separation was done on an ACQUITY UPLC BEH C18 column (100 mm × 2.1 mm, 1.7 μm) from Waters Corporation (Milford, MA, USA) using a mobile phrase of 0.1% methanoic acid in water (eluent A, *v*/*v*) and 0.1% methanoic acid in acetonitrile (eluent B, *v*/*v*) with a flow rate of 0.4 mL/min in a liner gradient program: 0–2 min, 10% B; 2–26 min, 10–90% B; 26–28 min, 90% B; 28–28.1 min, 90–10% B. The injection volume was 2 μL, the column temperature was maintained at 30 °C and the sample manager temperature was set at 15 °C.

#### 4.2.2. Experimental Design

The animal experimental procedures were conducted using Wistar rats (Male, 180~220 g) provided by Changchun Yisi experimental animal technology Co., Ltd. according to the protocols approved by the Review Committee of Animal Care and Use of Jilin University. The study was approved by the Institutional Animal Care and Use Committee of Jilin University School of Pharmaceutical Sciences (Approval No. 20200024). Prior to the experiment, the rats were housed at 20~25 °C under 12 h light/dark cycle with the relative humidity at 40~60%. After a habituation for 7 days, the animals were divided into six groups randomly (8 rats/group): sham group, model group, positive group (omeprazole, 4.0 mg/kg/day) and PPT groups of three doses (5.0, 10.0 and 20.0 mg/kg/day), namely the low-dose PPT group (L-PPT), moderate-dose PPT group (M-PPT) and high-dose PPT group (H-PPT) respectively, which was chosen based on the pre-experiments. All the rats were prohibited from food intaking for 16 h, and then the laparotomy was performed on all rats through a midline epigastric incision under anesthesia (10% chloral hydrate). The stomachs of all the rats were exposed, 0.3 mL of acetic acid was injected under mucosa at the junction of the stomach body and pyloric sinus for the rats in model group, omeprazole group and PPT treatment groups of three doses. And 0.3 mL of vehicle (0.9% saline) was injected into the stomachs of the rats in the sham group. During the operation the acetic acid was not allowed to escape. The stomach wound was carefully washed with 0.9% saline, and then cautiously put back to the abdominal cavity, embedded in the omentum, and the abdomen was finally sewed. Afterwards, the rats were subjected to the various treatments in a manner of gavage for 7 days. The rats were treated with intragastric administration of different samples once a day, and they freely had access to food and water supplies. The rats of sham and model groups were given 0.9% saline (10 mL/kg), the rats in the positive group were orally administered with omeprazole aqueous solution (0.4 mg/mL), and PPT treatment groups were with PPT aqueous solution (0.5, 1.0, 2.0 mg/mL). One hour after the last treatment, the rats were killed, and the stomach samples and blood samples were harvested.

#### 4.2.3. Preparation of Samples

Blood of all rats was collected 1 h after gavage from abdominal aorta and clotted at 4 °C for 1 h, then centrifuged at 3000 rpm for 10 min to obtain the serum. 800 µL serum was used to evaluate the ET-1, IL-6 and EGF levels. 500 µL serum was prepared for metabolomics analysis. Three times volume of methanol was added to the serum, then the supernatant was harvested by vortexing for 5 min, placing at 4 °C for 10 min and centrifugation at 10,000× *g* rpm for 10 min at 4 °C. After drying under a mild stream of nitrogen, the supernatant was dissolved with 80% methanol and 20% of water, and filtrated with a 0.22 µm syringe filter to prepare the test sample solution. Simultaneously, the quality control (QC) sample was prepared by mixing 20 μL of each sample.

#### 4.2.4. Therapeutic Effects

##### Body Weights

The body weights of the rats in all groups were measured every day.

##### Macroscopic Evaluation of Stomach

In order to evaluate the ulcer formations, after the animals were sacrificed, the stomach was collected and the abdomen was unfolded along the greater curvature. The stomach was opened and cleaned with 0.9% saline, and then to calculate the ulcer area (mm^2^).

##### Histological Analysis

The gastric mucosa was washed with phosphate buffer, fixed with 4% formaldehyde, dehydrated with gradient alcohol and embedded in paraffin. Then sections of 5 μm intervals were deparaffinized with xylene and ethanol, and stained with hematoxylin for 5 min, the residual dye solution was rinsed off with tap water. After treatment with eosin dye for 5 min, the sections were dehydrated by ethanol and xylene, and sealed with neutral gum. The general microstructure of the gastric mucosa was observed with microscope inspection, image acquisition and analysis.

##### Biochemical Estimations in Serum

The levels of endothelin-1 (ET-1), interleukin-6 (IL-6), epidermal growth factor (EGF), superoxide dismutase (SOD), malondialdehyde (MDA), tumor necrosis factor-α (TNF-α) in serum were evaluated using ELISA kits according to the instructions provided by the manufacturers. The ELISA steps of ET-1 and IL-6 were as follows: reconstituted biotinylated detection antibody and protein standard and diluted the wash buffer as specified. Performed serial dilution of protein standard and prepared samples as desired. Added 100 μL of protein standard, sample or control to each well and incubated for 2 h at room temperature. Aspirated protein standards, samples or controls out and washed plate 4 times. Diluted biotinylated detection antibody as specified, and added 100 μL to each well and incubated for 2 h at room temperature. Aspirated biotinylated detection antibody out and washed plate 4 times. Diluted streptavidin-HRP as specified. Added 100 μL of 1× Streptavidin-HRP to each well and incubated at room temperature for 60 min. Aspirated 1× Streptavidin-HRP out and washed plate 4 times. Added 100 μL of Ready-to-Use Substrate to each well and incubated at room temperature for color development. Add 100 μL of Stop Solution and read OD values at 450 nm. The ELISA steps of EGF were as follows: Prepared all reagents before starting assay procedure. Added 50 μL of Standard or Sample to the appropriate wells. Blank well didn’t add anyting. Added 100 μL of Enzymeconjugate to standard wells and sample wells except the blank well, covered with an adhesive strip and incubated for 60 min at 37 °C. Washed the Microtiter Plate 4 times. Added Substrate A 50 μL and Substrate B 50 μL to each well. Gently mixed and incubated for 15 min at 37 °C, protecting from light. Added 50 μL Stop Solution to each well. Read the OD values at 450 nm using a microtiter plate reader within 15 min.

##### Immunohistochemical Analysis

The gastric mucosa were stained immunohistochemically for ET-1 and EGFR protein expression. The paraffin-embedded sections were deparaffinized and incubated in methanol and 3% H_2_O_2_, and incubated overnight with ET-1 antibody and EGFR antibody, respectively. Sections were then stained using avidin-biotin-peroxidase complex method and counterstained with hematoxylin. Image-Pro-Plus software(IPP) 6.0 was utilized to evaluate the intensity of the immunohistochemistry. Firstly, the intensity calibration of the image was done by choosing the brightest pixels. Secondly, the area of interest (AOI) was selected manually and the measurement options were settled. Finally, the area of the AOI was measured followed by the collection of the Integral Optical Density Values (IOD values). OD value was calculated by IOD/area. Each group was evaluated with 3 different AOI and came out with the average and the standard deviation of OD values.

#### 4.2.5. Metabolomics Study

##### Method Validation of Metabolomics Study

The method was validated in system stability, precision, reproducibility and sample stability. Firstly, the system stability was accessed by QC sample, which was randomly run covering the whole analysis process, and eight exact mass/retention time pairs were selected from different spectral regions in ESI+ and ESI- respectively. Secondly, the precision of the method was evaluated by the QC sample with six consecutive replicate injections. Thirdly, the reproducibility of sample preparation in this method was investigated by performing six parallel replicates of a chosen sample. Fourthly, the sample stability of those analytes was tested by analyzing 1 serum sample at 4 °C for 0, 4, 8, 10, and 12 h. The results of validation were expressed as the relative standard deviation (RSD).

##### Data Analysis of Metabolomics Strategies

The raw file of the original mass spectrometry data was acquired and processed in MarkerLynx XS Version 4.1 software, with the optimized parameters as follows: retention time, 2~28 min; retention time window, 0.20; mass, 50~1200 Da; mass tolerance, 0.10 ppm; mass window, 0.10 ppm; noise elimination, level 6. Then SIMCA-P sofware (Version 14.1, Umetric, Umea, Sweden) was used for multivariate data analysis. Principle component analysis (PCA) was performed to generate an overview for clustering of different groups and to search for potential outliers. Orthogonal projections to latent structures discriminant analysis (OPLS-DA) was then carried out to evaluate the maximum separation between two different groups. The corresponding S-plots were used to distinguish spectral variables and provide visual presentation of the OPLS-DA predictive results that contributed to the separation of the samples. The quality was also validated with R2 and Q2 followed by rigorous permutation tests (number: 200) to indicate statistical significance. The differential metabolites with *p*-value below 0.05 and variable importance in the projection (VIP) greater than 1.0 were considered potential biomarkers. Then, the potential biomarkers were also screened with the area under curve (AUC) > 0.8 in the predictive receiver operating characteristic (ROC) curves. Further, the biomarkers were identified by comparing with either the standards or the tandem mass spectrometry (MS/MS) fragmentation patterns based on HMDB and METLIN databases. Finally, the MetaboAnalyst 5.0 was used to confirm potential metabolites and metabolic pathways (the impact-value > 0.10).

#### 4.2.6. Network Pharmacology Analysis

In order to reveal the key targets and related proteins and pathways, and provide visualization of the component-targets-pathways, Cytoscape 3.8.2 (Cytoscape Consortium, San Diego, CA, USA) was used to construct the network. Firstly, the disease targets of GU were searched in GeneCards (https://www.genecards.org/), DrugBank (http://www.drugbank.ca/), GAD (http://geneticassociationdb.nih.gov/), OMIM (http://www.omim.org/), Disgenet (https://www.disgenet.org/), TTD (http://database.idrb.cqu.edu.cn/TTD/) and other databases by using ‘gastric ulcer’ or ‘stomach ulcer’ as the keyword. Secondly, the molecular targets of PPT were filtered by searching the keywords ‘Protopanaxatriol’ from PharmMapper (http://lilab-ecust.cn/pharmmapper/), SEA database (http://sea.bkslab.org), STITCH (http://stitch.embl.de/), SwissTarget (http://www.swisstargetprediction.ch/), BATMAN-TCM (http://bionet.ncpsb.org.cn/batman-tcm/). Thirdly, the intersection of compound targets and disease targets was considered the predicted targets of PPT against GU, which was used to screen core targets. And the Cytoscape 3.8.2 software was used to construct the visual regulation network diagram of “component-targets-disease”. Fourthly, a protein-protein interaction (PPI) network was created by uploading the core target data to the String (http://string-db.org/cgi/input.pl) database and hiding the discrete nodes (highest confidence = 0.9) via Cytoscape 3.8.2 software. Then the hub gene network was constructed with the hub genes which were screened from the key nodes (Degre e ≥ 7) in the PPI network. Fifthly, the Gene Ontology (GO) enrichments and Kyoto Encyclopedia of Genes and Genomes (KEGG) analysis were performed. Uploaded the core target data to the Gprofiler (https://biit.cs.ut.ee/gprofiler/convert) database for GO analysis and download the data file. The GO enrichment histogram was obtained through GO visualization processing with OmicShare Tools (https://www.omicshare.com/tools/). Then the core target data was uploaded to the DAVID (https://david.ncifcrf.gov/) database for KEGG Pathway analysis. We identified key pathways by combining evidences from the literature. The bubble chart of KEGG enrichment was obtained through visualizing the pathways with Omicshare (https://auth.lifemapsc.com/). Base on the bubble chart, Cytoscape 3.8.2 was used to construct the visual regulation network diagram of ‘component-targets-pathways’.

#### 4.2.7. Integrated Analysis Involving Metabolomics and Network Pharmacology

In order to further confirm the key potential metabolites, targets and metabolic pathways, metabolomics and network pharmacology were merged to construct an integrative network. The potential metabolites (identified in metabolomics) and key targets (identified in network pharmacology) were imported into the MetScape plugin to construct the ‘biomarker-reaction-enzyme-target’ network, then the key biomarkers, targets and metabolic pathways could be recognized.

#### 4.2.8. Molecular Docking

Molecular docking was carried out to predict the binding modes of key targets with PPT using Schrödinger (Version 6.7, LLC, New York, NY, USA, 2015). Firstly, the crystal structures of key targets screened out in network pharmacology were retrieved from the RCSB Protein Data Bank (https://www.rcsb.org/). The receptors were prepared using the Protein Preparation Wizard in the GLIDE software. Secondly, the ligand, namely PPT, was drawn with Maestro Elements and subjected to minimization using the OPLS3 force field. Thirdly, the Receptor Grid Generation and GLIDE docking were carried out. Extra precision docking was conducted, and the parameters of partial charge cutoff and scaling factor were set at the default values 0.15 and 0.80, respectively. PyMOL was used to produce the final figures of the docking results.

## 5. Conclusions

In this study, we have demonstrated that PPT has therapeutic effects in acetic-acid-induced GU rats. Serum metabolomics elucidated that 26 differential metabolites involved in 7 metabolic pathways were recognized as the biomarkers of the anti-GU effect of PPT. An integrated strategy based on metabolomics and network pharmacology was developed to decipher the key targets and mechanisms of PPT in treating GU. The combined analysis revealed three key targets (CYP2C9, CYP3A4 and PIK3CA), as well as related metabolites and pathways. The targets were further validated by molecular docking. This research shed light on the pathogenesis of GU and provides a promising anti-ulcer candidate.

## Figures and Tables

**Table 1:** The RSDs (%) of peak intensity and RT in validation tests.

Tests	ESI+ Mode		ESI- Mode	
	Peak Intensity	RT	Peak Intensity	RT
System stability	0.38–4.19	0.24–1.75	0.56–1.94	0.16–1.13
Precision	0.35–3.41	0.10–0.41	0.19–4.65	0.07–0.68
Reproducibility	0.06–0.39	0.94–1.17	0.14–0.67	1.07–1.93
Sample stability	1.07–4.47	0.07–0.98	0.97–2.96	0.10–1.75

The above validation results revealed that system stability, precision, reproducibility, and sample stability of the UPLC-QTOF-MS method were reliable, which could be used for analyzing the tested samples.

**Table 2:** The AUCs and *p* values of the biomarkers in different predictive ROC curves.

Compound No.	Sham & Model		Model & H-PPT		Compound No.	Sham & Model		Model & H-PPT	
	AUC	*p*	AUC	*p*		AUC	*p*	AUC	*p*
1	0.922	0.005	0.922	0.005	14	1.000	0.001	1.000	0.001
2	1.000	0.001	1.000	0.001	15	0.938	0.001	1.000	0.001
3	1.000	0.001	1.000	0.001	16	1.000	0.001	1.000	0.001
4	0.984	0.001	0.984	0.001	17	1.000	0.001	1.000	0.001
5	1.000	0.001	1.000	0.001	18	0.984	0.001	0.984	0.001
6	0.984	0.001	0.906	0.006	19	1.000	0.001	1.000	0.001
7	0.922	0.005	0.938	0.003	20	0.984	0.001	1.000	0.001
8	0.938	0.003	0.922	0.005	21	1.000	0.001	1.000	0.001
9	1.000	0.001	1.000	0.001	22	0.953	0.002	0.984	0.001
10	1.000	0.001	0.922	0.005	23	1.000	0.001	0.953	0.002
11	1.000	0.001	1.000	0.001	24	0.906	0.006	0.953	0.002
12	1.000	0.001	1.000	0.001	25	0.969	0.002	0.875	0.002
13	0.953	0.002	0.891	0.009	26	0.922	0.005	0.984	0.001

**Table 3:** Distinct metabolites identified in serum samples.

No.	RT	Mass	Compound Name	VIP	Formula	Adducts	Δm	HMDB ID	Pathways	Content Level
1 *	0.60	203.0542	Paraxanthine	2.42	C_7_H_8_N_4_O_2_	M+Na	−1.48	0001860	CM	M < H-PPT
2 ^a^	10.99	774.6042	PE(22:1(13Z)/16:0)	1.68	C_43_H_84_NO_8_P	M+H	3.74	0009517	GM	M > H-PPT
3 ^a^	11.07	811.6660	SM(d18:0/22:0)	2.37	C_45_H_93_N_2_O_6_P	M+Na	−1.11	0012091	SM	M < H-PPT
4 ^a^	11.23	752.5573	PE(20:3(8Z,11Z,14Z)/P-18:1(9Z))	2.00	C_43_H_78_NO_7_P	M+H	−2.79	0009381	GM	M > H-PPT
5 ^a^	11.24	892.2159	(2*E*)-Octenoyl-CoA	2.48	C_29_H_48_N_7_O_17_P_3_S	M+H	4.60	0003949	FAE	M > H-PPT
6 ^a^	11.25	783.6333	SM(d18:0/20:0)	2.31	C_43_H_89_N_2_O_6_P	M+Na	−2.94	0012090	SM	M < H-PPT
7 ^a^	11.27	777.5883	SM(d18:0/20:2(11Z,14Z))	2.70	C_43_H_83_N_2_O_6_P	M+Na	−0.39	0013465	SM	M < H-PPT
8 ^a^	11.36	821.6523	SM(d17:1/24:1(15Z))	3.09	C_46_H_91_N_2_O_6_P	M+Na	1.34	0011696	SM	M < H-PPT
9 ^a^	11.37	958.2241	3-Oxodecanoyl-CoA	2.11	C_31_H_52_N_7_O_18_P_3_S	M+Na	4.28	0003939	FAE	M > H-PPT
10 ^a^	11.39	972.7340	Lactosylceramide(d18:1/24:1(15Z))	1.46	C_54_H_101_NO_13_	M+H	−1.13	0004872	SM	M > H-PPT
11 ^a^	11.46	675.5469	SM(d18:1/14:0)	1.51	C_37_H_75_N_2_O_6_P	M+H	4.14	0012097	SM	M < H-PPT
12 ^a^	11.49	890.6626	Lactosylceramide (d18:1/18:0)	2.00	C_48_H_91_NO_13_	M+H	4.49	0011591	SM	M > H-PPT
13 ^a^	11.54	922.2543	Decanoyl-CoA (n-C10:0CoA)	2.28	C_31_H_54_N_7_O_17_P_3_S	M+H	−4.88	0006404	FAE	M > H-PPT
14 ^a^	11.61	966.2805	(*S*)-3-Hydroxydodecanoyl-CoA	1.66	C_33_H_58_N_7_O_18_P_3_S	M+H	−4.66	0003936	FAE	M > H-PPT
15 ^a^	12.39	355.2495	Prostaglandin F2a	2.48	C_20_H_34_O_5_	M+H	3.09	0001139	AM	M > H-PPT
16 *	12.72	318.3010	Phytosphingosine	5.59	C_18_H_39_NO_3_	M+H	0.63	0004610	SM	M < H-PPT
17 *	14.94	302.3054	Sphinganine	2.46	C_18_H_39_NO_2_	M+H	−1.65	0000269	SM	M < H-PPT
18 ^a^	16.09	544.3398	LysoPC(18:1(9*Z*))	13.28	C_26_H_52_NO_7_P	M+Na	3.49	0002815	GM	M > H-PPT
19 *	17.08	295.2279	12,13-EpOME	1.23	C_18_H_32_O_3_	M-H	2.03	0004702	LM	M > H-PPT
20 *	18.11	319.2276	19(*S*)-HETE	6.00	C_20_H_32_O_3_	M-H	0.94	0004680	AM	M > H-PPT
21 ^a^	20.54	524.3709	PC(O-16:0/2:0)	26.76	C_26_H_54_NO_7_P	M+H	−1.33	0062195	EM	M > H-PPT
22 ^a^	21.15	510.3917	LysoPC(O-18:0)	1.46	C_26_H_56_NO_6_P	M+H	−1.37	0011149	EM	M > H-PPT
23 *	22.81	303.2327	Arachidonic acid	4.54	C_20_H_32_O_2_	M-H	0.99	0001043	AM	M > H-PPT
24 *	23.14	279.2325	Linoleic acid	1.71	C_18_H_32_O_2_	M-H	3.96	0000673	LM	M < H-PPT
25 ^a^	26.33	813.6753	SM(d18:1/24:1(15Z))	1.95	C_47_H_93_N_2_O_6_P	M+H	0.37	0012107	SM	M < H-PPT
26 ^a^	26.39	830.5443	3-*O*-Sulfogalactosylceramide (d18:1/18:0)	1.63	C_42_H_81_NO_11_S	M+Na	1.81	0012314	SM	M < H-PPT

RT, Retention Time, min; Mass, Measured mass, Da; Δm, Relative deviation, ppm; * Metabolites validated with standards; ^a^ Metabolites confirmed by MS/MS fragments and the predicted MS/MS spectra in database of HMDB or METLIN; “M” represents model group; “PC”: Phosphatidylcholine; “LysoPC”: Lysophosphatidyl- choline; “HETE”: Hydroxyeicosatetraenoic acid; SM: Sphingomyelin.

**Table 4:** The results from the metabolic pathways of differential metabolites.

Pathway Name	Match Status	*p*	−log (*p*)	Holm *p*	FDR	Impact
Sphingolipid metabolism	5/21	2.397 × 10^−6^	5.6203	2.0135 × 10^−4^	2.0135 × 10^−4^	0.1582
Linoleic acid metabolism	3/5	0.0013	2.8806	0.1066	0.0276	1.000
Fatty acid elongation	4/27	4.2911 × 10^−4^	7.7538	0.0339	0.0116	0.1075
Arachidonic acid metabolism	3/36	0.0080	2.0991	0.6367	0.1337	0.3329
Ether lipid metabolism	2/13	0.2146	0.6684	1.0000	1.0000	0.1446
Glycerophospholipid metabolism	2/30	0.0667	1.1762	1.0000	0.7998	0.1219
Caffeine metabolism	1/12	0.1348	0.8710	1.0000	1.0000	0.6923

**Table 5:** The information of key targets and pathways.

No.	Target	PDB ID	Bind Energy (Kcal/mol)	Related Pathway
1	CYP2C9	1R9O	−6.78	AM, LM
2	CYP3A4	2V0M	−9.40	LM
3	PIK3CA	3ZIM	−9.27	SM

## Supplementary Materials

The following supporting information can be downloaded at: https://www.mdpi.com/article/10.3390/ijms232012097/s1.

## Abbreviations

ANOVA	Analysis of variance
AUC	Area under curve
EGF	Epidermal growth factor
EGFR	Epidermal growth factor receptor
ELISA	Enzyme-linked immunosorbent assay
ESI	Electrospray ionization
ET-1	Endothelin-1
GU	Gastric ulcer
H&E	Hematoxylin and eosin
HETE	Hydroxyeicosatetraenoic acid
H-PPT	High-dose PPT
L-PPT	Low-dose PPT
LysoPC	Lysophosphatidylcholine
M-PPT	Moderate-dose PPT
MDA	Malondialdehyde
OPLS-DA	Orthogonal Projections to Latent Structures Discriminant Analysis
PC	Phosphatidylcholine
PCA	Principal Component Analysis
PDB	Protein Data Bank
QC	Quality Control
QTOF-MS	Quadrupole Time of Flight-Mass Spectrometry
ROC	Receiver operating characteristic
RSD	Relative Standard Deviation
RT	Retention Time
SD	Standard deviation
SM	Sphingomyelin
SOD	Superoxide dismutase
TNF-α	Tumor necrosis factor-α
UPLC	Ultra-Performance Liquid Chromatography
VIP	Variable importance in the projection
