# Supplementary material for: Integrated Metabolomics and Network Pharmacology to Decipher the Latent Mechanisms of Protopanaxatriol against Acetic Acid-Induced Gastric Ulcer
Source: Int J Mol Sci. 2022 Oct 11;23(20):12097. doi: 10.3390/ijms232012097 (PMC9602736; doi:10.3390/ijms232012097)
Supplement: Supplementary file 1 [file ijms-23-12097-s001.zip › ijms-1788930-supplementary.pdf]

Supplementary Material

PART I: The superimposed view of the representative base peak intensity (BPI) chromatograms

PART II: The box plot of potential biomarkers changes

PART III: The Information of the Key targets

PART IV: The “component-targets-disease” network and hub gene network.

PART I: The superimposed view of the representative base peak intensity (BPI) chromatograms

(A)

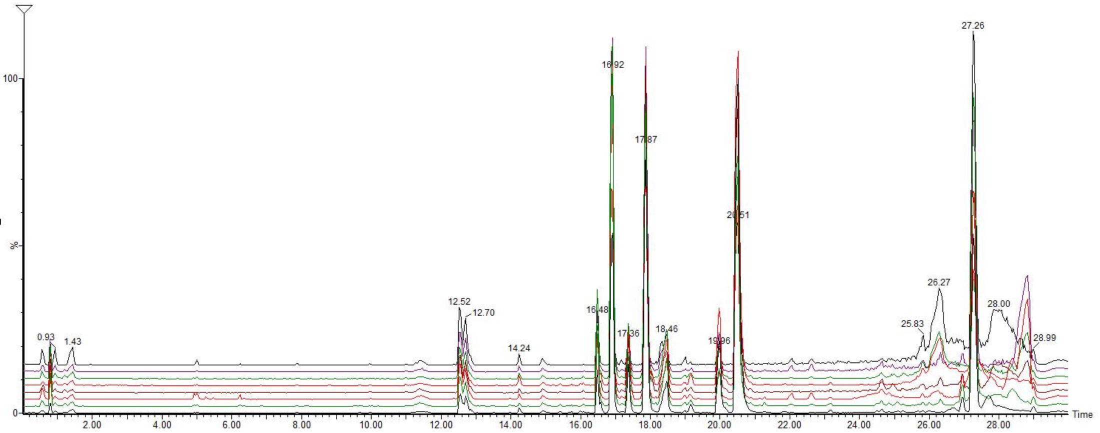

(B)

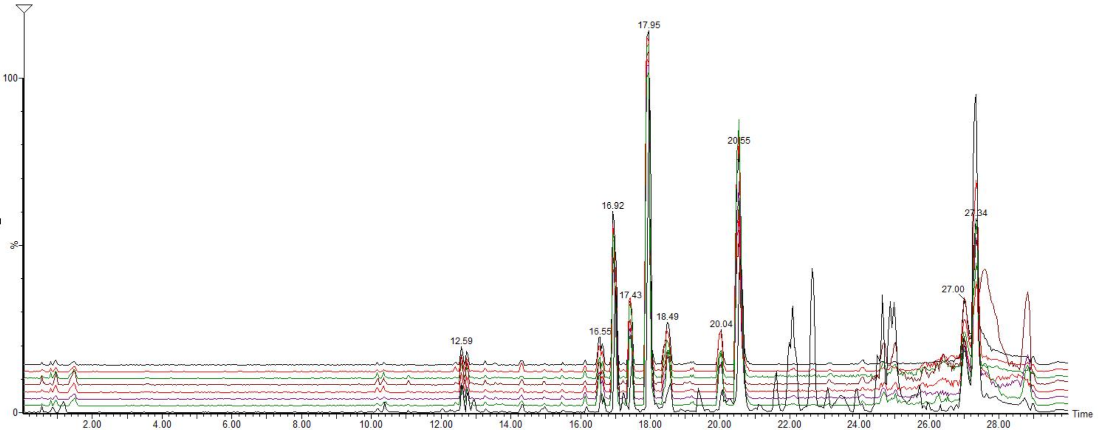

(C)

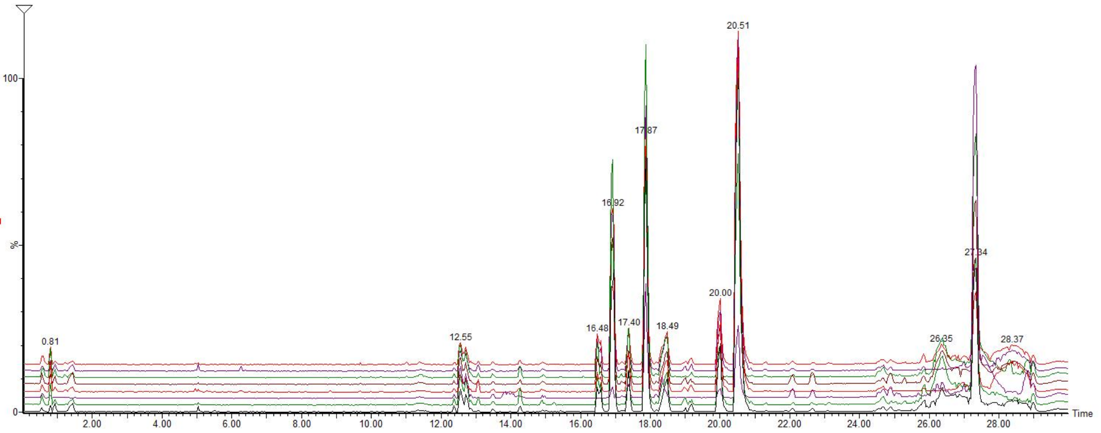

(D)

(E)

(F)

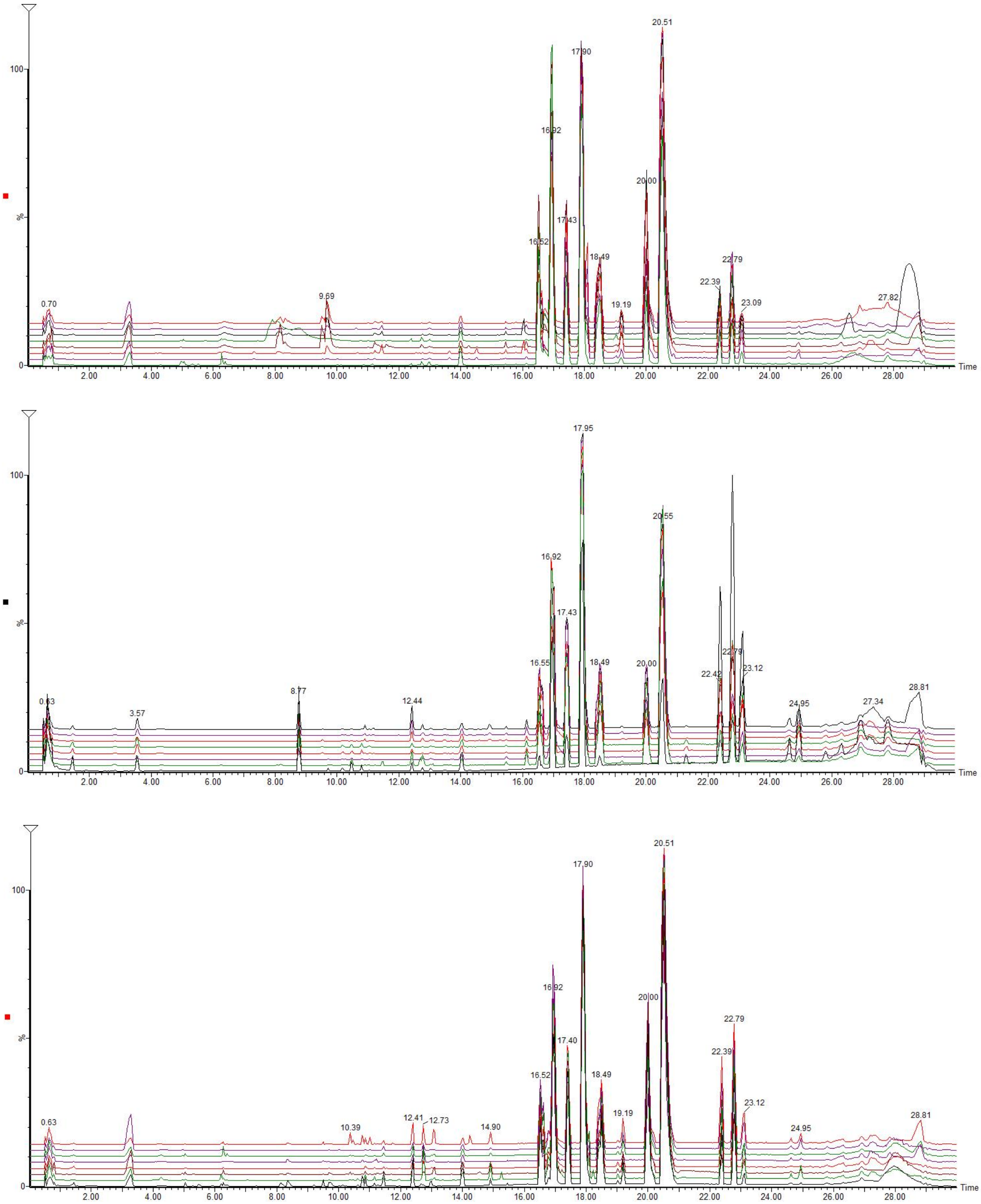

Figure S1. The superimposed view of the representative base peak intensity (BPI) chromatograms of serum samples of normal **(A)**, model **(B)** and H-PPT **(C)** groups in positive modes; and those of normal **(D)**, model **(E)** and H-PPT **(F)** groups in negative mode.

PART II: The box plot of potential biomarkers changes

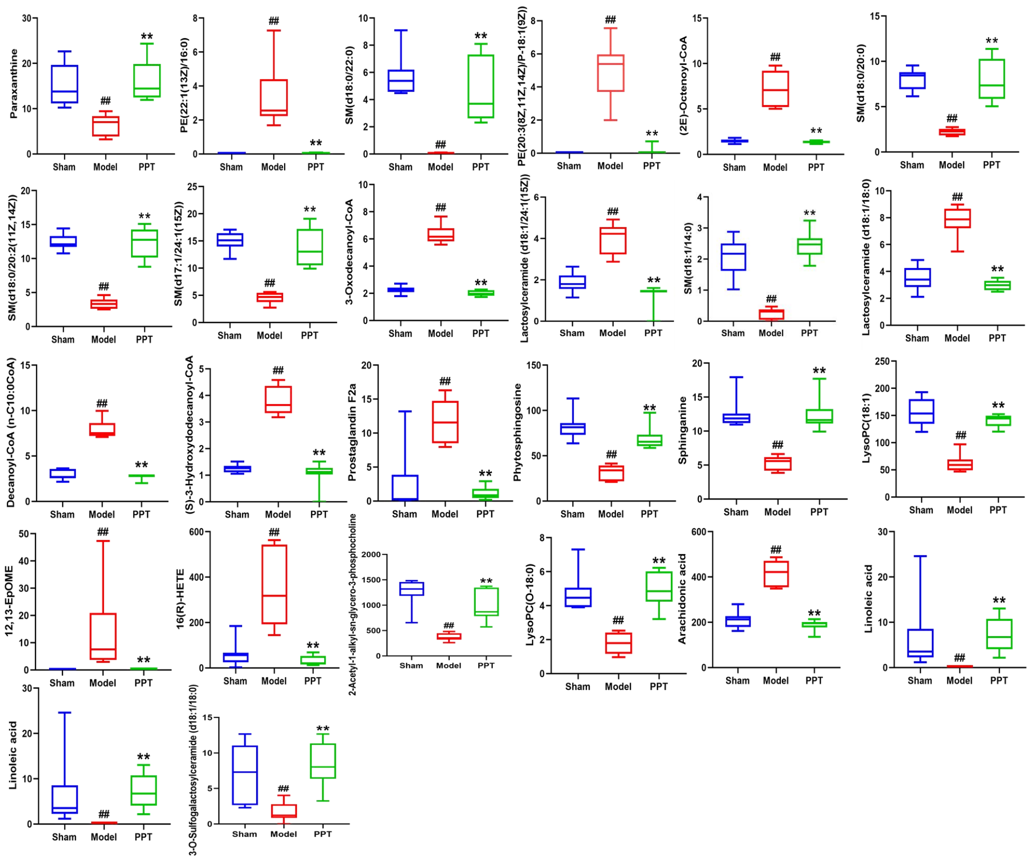

Figure S2. The box plot of potential biomarkers changes in GU with PPT treatment (n=8). The comparison between two groups was completed by *t*-test. (Compared with sham group, ## $p < 0.01$; compared with model group, ** $p < 0.01$).

PART III: The Information of the Key targets

Table S1. The Information of the Key targets

No	Protein Name	Gene Name	Type	UniProt ID	Degree	Betweenness	Database for targets of PPT	Database for targets of GU
1	BCL2 Associated X, Apoptosis Regulator	BAX	Cytokin	Q07812	6	0.0050	BATMAN	GeneCards
2	Cyclin-T1	CCNT1	Cytokin	O60563	3	0	Swiss	GeneCards
3	Fibroblast growth factor 23	FGF23	Cytokin	Q9GZV9	12	0.0076	BATMAN	GeneCards
4	Mediator Complex Subunit 1	MED1	Cytokin	Q15648	13	0.0205	BATMAN	GeneCards
5	Signal transducer and activator of transcription 3	STAT3	Cytokin	P40763	27	0.0885	Swiss	GeneCards
6	Transcription Factor 3	TCF3	Cytokin	P15923	5	0.0015	BATMAN	GeneCards
7	Acetylcholinesterase	ACHE	Enzyme	P22303	4	0.0012	Swiss	GeneCards
8	ALK tyrosine kinase receptor	ALK	Enzyme	Q9UM73	13	8.86E-04	Swiss	GeneCards
9	Potassium-transporting ATPase alpha chain 2	ATP12A	Enzyme	P54707	10	0.0155	Swiss	GeneCards
10	Aurora kinase A	AURKA	Enzyme	O14965	6	0	Swiss	GeneCards
11	Beta-1,4-galactosyltransferase 1	B4GALT1	Enzyme	P15291	0	0	BATMAN	GeneCards
12	Butyrylcholinesterase	BCHE	Enzyme	P06276	3	0	Swiss	GeneCards
13	Calpain-2 catalytic subunit	CAPN2	Enzyme	P17655	3	6.20E-04	Swiss	GeneCards
14	Cathepsin D	CTSD	Enzyme	P07339	7	0.0018	Swiss	GeneCards
15	Cathepsin E	CTSE	Enzyme	P14091	0	0	Swiss	GeneCards
16	Cytochrome P450 Family 19 Subfamily A Member 1	CYP19A1	Enzyme	P11511	19	0.0472	Swiss	GeneCards
17	Cytochrome P450 Family 27 Subfamily B Member 1	CYP27B1	Enzyme	O15528	14	0.0165	BATMAN	GeneCards
18	Cytochrome P450 Family 2 Subfamily C Member 19	CYP2C19	Enzyme	P33261	16	0.0317	Swiss	DisGeNET
19	Cytochrome P450 Family 2 Subfamily C Member 9	CYP2C9	Enzyme	P11712	15	0.0299	Swiss	DisGeNET
20	Cytochrome P450 Family 2 Subfamily D Member 6	CYP2D6	Enzyme	P10635	15	0.0233	Swiss	GeneCards
21	Cytochrome P450 Family 3 Subfamily A Member 4	CYP3A4	Enzyme	P08684	25	0.0790	Swiss	GeneCards
22	Cytochrome P450 Family 51 Subfamily A Member 1	CYP51A1	Enzyme	Q16850	5	0.0119	Swiss	GeneCards
23	Epoxide Hydrolase 2	EPHX2	Enzyme	P34913	2	0	Swiss	GeneCards
24	Erb-B2 Receptor Tyrosine Kinase 2	ERBB2	Enzyme	P04626	27	0.0252	Swiss	GeneCards
25	Fibroblast Growth Factor Receptor 1	FGFR1	Enzyme	P11362	18	0.0134	Swiss	GeneCards
26	3-Hydroxy-3-Methylglutaryl-CoA Reductase	HMGCR	Enzyme	P04035	11	0.0239	Swiss	GeneCards
27	Corticosteroid 11-beta-dehydrogenase isozyme 1	HSD11B1	Enzyme	P28845	8	0.0084	Swiss	GeneCards

28	Corticosteroid 11-beta-dehydrogenase isozyme 2	HSD11B2	Enzyme	P80365	3	2.26E-04	SEA	GeneCards
29	Insulin Receptor	INSR	Enzyme	P06213	15	0.0114	Swiss	GeneCards
30	Janus Kinase 1	JAK1	Enzyme	P23458	17	0.0047	Swiss	GeneCards
31	Janus Kinase 2	JAK2	Enzyme	O60674	20	0.0221	Swiss	GeneCards
32	Kinase Insert Domain Receptor	KDR	Enzyme	P35968	19	0.0196	Swiss	DisGeNET
33	KIT Proto-Oncogene, Receptor Tyrosine Kinase	KIT	Enzyme	P10721	19	0.0078	Swiss	GeneCards
34	LIM Domain Kinase 1	LIMK1	Enzyme	P53667	1	0	Swiss	GeneCards
35	Lanosterol Synthase	LSS	Enzyme	P48449	4	0.0012	SEA	GeneCards
36	Mitogen-Activated Protein Kinase 14	MAPK14	Enzyme	Q16539	17	0.0195	Swiss	GeneCards
37	Mitogen-Activated Protein Kinase 8	MAPK8	Enzyme	P45983	17	0.0125	Swiss	GeneCards
38	MDM2 Proto-Oncogene	MDM2	Enzyme	Q00987	19	0.0064	Swiss	GeneCards
39	Mechanistic Target Of Rapamycin Kinase	MTOR	Enzyme	P42345	29	0.0506	Swiss	GeneCards
40	Phosphodiesterase 4B	PDE4B	Enzyme	Q07343	0	0	Swiss	GeneCards
41	Phosphatidylinositol-4,5-Bisphosphate 3-Kinase Catalytic Subunit Alpha	PIK3CA	Enzyme	P42336	28	0.0257	Swiss	GeneCards
42	Phosphatidylinositol-4,5-Bisphosphate 3-Kinase Catalytic Subunit Beta	PIK3CB	Enzyme	P42338	16	0.0041	Swiss	GeneCards
43	Phosphatidylinositol-4,5-Bisphosphate 3-Kinase Catalytic Subunit Delta	PIK3CD	Enzyme	O00329	14	0.0018	Swiss	GeneCards
44	Phosphatidylinositol-4,5-Bisphosphate 3-Kinase Catalytic Subunit Gamma	PIK3CG	Enzyme	P48736	11	0.0103	Swiss	GeneCards
45	Protein Phosphatase 2 Catalytic Subunit Alpha	PPP2CA	Enzyme	P67775	8	0.0069	Swiss	GeneCards
46	Prostaglandin E Synthase	PTGES	Enzyme	O14684	3	5.70E-04	Swiss	GeneCards
47	Prostaglandin-Endoperoxide Synthase 1	PTGS1	Enzyme	P23219	6	0.0058	Swiss	DisGeNET
48	Prostaglandin-Endoperoxide Synthase 2	PTGS2	Enzyme	P35354	24	0.0951	Swiss	DisGeNET
49	Protein Tyrosine Phosphatase Non-Receptor Type 1	PTPN1	Enzyme	PTPN1	12	0.0099	Swiss	GeneCards
50	Renin	REN	Enzyme	P00797	15	0.0654	Swiss	GeneCards
51	Sterol O-Acyltransferase 1	SOAT1	Enzyme	P35610	2	0	SEA	GeneCards
52	UDP Glucuronosyltransferase Family 2 Member B7	UGT2B7	Enzyme	P16662	10	0.0085	Swiss	GeneCards
53	ATP Binding Cassette Subfamily B Member 11	ABCB11	Other	O95342	11	0.0075	SEA	GeneCards
54	Klotho	KL	Other	Q9UEF7	11	0.0158	BATMAN	GeneCards
55	Solute Carrier Family 6 Member 4	SLC6A4	Other	P31645	12	0.0380	Swiss	GeneCards
56	Crystallin Alpha B	CRYAB	Protein	P02511	3	0	SEA	GeneCards
57	GC Vitamin D Binding Protein	GC	Protein	P02774	8	0.0027	BATMAN	GeneCards

58	Heat Shock Protein 90 Alpha Family Class A Member 1	HSP90AA1	Protein	P07900	29	0.0977	Swiss	GeneCards
59	Sodium Voltage-Gated Channel Alpha Subunit 9	SCN9A	Protein	Q15858	2	0.0022	Swiss	GeneCards
60	Sex Hormone Binding Globulin	SHBG	Protein	P04278	7	0.0025	Swiss	GeneCards
61	Wnt Family Member 4	WNT4	Protein	P56705	4	0	BATMAN	GeneCards
62	Adenosine A1 Receptor	ADORA1	Receptor	P30542	4	0.0085	Swiss	GeneCards
63	Adenosine A2a Receptor	ADORA2A	Receptor	P29274	6	0.019	Swiss	GeneCards
64	Androgen Receptor	AR	Receptor	P21730	18	0.0137	Swiss	GeneCards
65	C-C Motif Chemokine Receptor 1	CCR1	Receptor	P32246	3	0.0241	Swiss	GeneCards
66	Corticotropin Releasing Hormone Receptor 1	CRHR1	Receptor	P34998	5	0.0020	Swiss	GeneCards
67	Dopamine Receptor D2	DRD2	Receptor	P14416	7	0.0086	Swiss	GeneCards
68	Estrogen Receptor 1	ESR1	Receptor	P03372	33	0.0964	Swiss	GeneCards
69	Estrogen Receptor 2	ESR2	Receptor	Q92731	14	0.0106	Swiss	GeneCards
70	Coagulation Factor II Thrombin Receptor	F2R	Receptor	P25116	3	0.0013	Swiss	GeneCards
71	G Protein-Coupled Bile Acid Receptor 1	GPBAR1	Receptor	GPBAR1	5	4.41E-04	BATMAN	GeneCards
72	G Protein-Coupled Receptor 18	GPR18	Receptor	Q14330	1	0	Swiss	GeneCards
73	G Protein-Coupled Receptor 55	GPR55	Receptor	Q9Y2T6	3	0.0244	Swiss	GeneCards
74	5-Hydroxytryptamine Receptor 1A	HTR1A	Receptor	P08908	9	0.0212	Swiss	GeneCards
75	Interleukin 6 Cytokine Family Signal Transducer	IL6ST	Receptor	P40189	7	1.07E-04	Swiss	
76	Integrin Subunit Alpha L	ITGAL	Receptor	P20701	1	0	Swiss	GeneCards
77	Nuclear Receptor Subfamily 1 Group H Member 3	NR1H3	Receptor	Q13133	4	1.27E-04	Swiss	GeneCards
78	Nuclear Receptor Subfamily 1 Group H Member 4	NR1H4	Receptor	Q96RI1	12	0.022	BATMAN	GeneCards
79	Nuclear Receptor Subfamily 1 Group I Member 2	NR1I2	Receptor	O75469	11	0.0165	Swiss	GeneCards
80	Nuclear Receptor Subfamily 1 Group I Member 3	NR1I3	Receptor	Q14994	10	0.0081	Swiss	GeneCards
81	Opioid Receptor Delta 1	OPRD1	Receptor	P41143	3	0.0012	Swiss	GeneCards
82	Opioid Receptor Mu 1	OPRM1	Receptor	P35372	8	0.0363	Swiss	GeneCards
83	Purinergic Receptor P2X 3	P2RX3	Receptor	P56373	3	0.0011	Swisst	GeneCards
84	Progesterone Receptor	PGR	Receptor	Q9Y605	22	0.0403	BATMAN	GeneCards
85	RAR Related Orphan Receptor C	RORC	Receptor	P51449	1	0	Swiss	GeneCards
86	Retinoid X Receptor Alpha	RXRA	Receptor	P19793	13	0.0139	BATMAN	GeneCards
87	Smoothened, Frizzled Class Receptor	SMO	Receptor	Q99835	0	0	Swiss	GeneCards
88	Tachykinin Receptor 2	TACR2	Receptor	P21452	0	0	Swiss	GeneCards
89	Vitamin D Receptor	VDR	Receptor	P11473	13	0.0212	Swiss	GeneCards

PART IV: The “component-targets-disease” network and hub gene network

The “component-targets-disease” network and hub gene network are shown in Figure S3 and Figure S4.

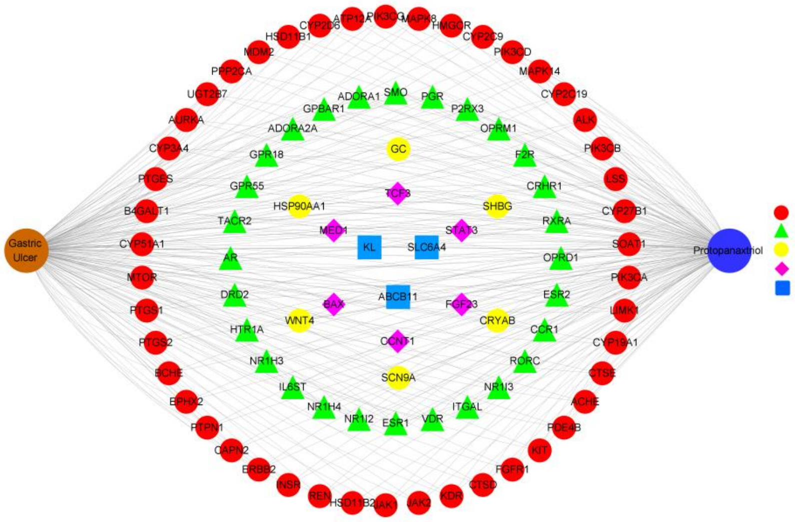

Figure S3. The“component-targets-disease”network.

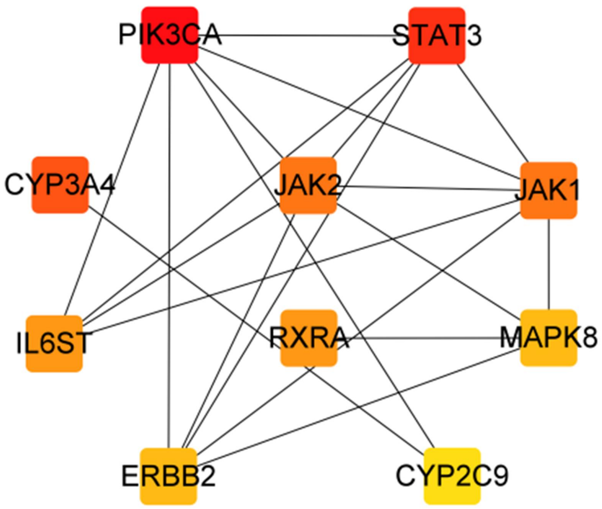

Figure S4. The hub gene network.
